# Combined Rigid-Flexible Multibody Analysis Reveals Reduced Pedicle Screw Loads in Short-Segment Fixation for Decompressed Lumbar Spine Stabilization

**DOI:** 10.1007/s10439-025-03706-1

**Published:** 2025-03-13

**Authors:** Simone Borrelli, Giovanni Putame, Stefano Marone, Andrea Ferro, Alberto L. Audenino, Mara Terzini

**Affiliations:** 1 PolitoBIOMed Lab, Politecnico di Torino, Turin, Italy; 2 Department of Mechanical and Aerospace Engineering, Politecnico di Torino, Turin, Italy; 3 Oncologic Orthopaedic Surgery Division, CTO Hospital – Città della Salute e della Scienza di Torino, Turin, Italy

**Keywords:** Pedicle screw, Short-segment fixation, Long-segment fixation, Spinal fixation, Spinal metastasis, Multibody model, Flexible body modelling, Spinal cord decompression, Coupled modelling, Fatigue strength

## Abstract

**Background:**

Spinal cord compression in patients with vertebral metastases often requires surgical decompression with spinal fixation. Recent studies reported increased implant failures due to mechanical complications, raising concerns about current clinical practices. Long-segment fixation (*Lf*) is commonly employed to enhance mechanical stability and reduce the severity of pedicle screw failure. The study investigates how the number of vertebral levels involved in fixation affects the loads on pedicle screw anchorages in a fatigue-related displacement domain.

**Method:**

Using a rigid-flexible multibody approach, a non-linear T12–S1 model was employed to simulate two fixation types following L3 posterior decompression surgery: *Lf* spanning two levels above and below the decompression site (L1, L2, L4, and L5) and a short-segment fixation (*Sf*) involving only adjacent vertebrae. Internal reactions at the rod-pedicle screw anchorages were estimated in terms of pullout, shear forces, and bending moments. The range of motion analysed (flexion: 22°, extension: 8°, lateral bending: 12°, axial rotation: 5°) was confined to the “Cone of Economy”, representing a small-displacement volume where loads are assumed cyclically exchanged.

**Results:**

*Lf* exhibited up to fivefold higher reactions than *Sf*, with a heterogeneous shear force distribution: middle screws appeared shielded, while extremity screws were overloaded (~400 N, comparable to experimental fatigue strength). Pullout forces remained within safe limits (< 150 N).

**Conclusions:**

The rigid-flexible multibody approach effectively estimated internal loads in the implant-spine constructs under dynamic conditions. The findings highlight the long-term implications of *Lf*, demonstrating that involving more vertebral levels triggers adverse loads on pedicle screws, potentially compromising implant durability.

**Supplementary Information:**

The online version contains supplementary material available at 10.1007/s10439-025-03706-1.

## Introduction

Spine is among the most common sites for metastatic disease, with the thoracolumbar segment being predominantly affected [1]. Vertebral metastases often lead to severe complications, such as spinal cord compression, which requires surgical decompression to relieve neurological symptoms and pain [2, 3]. However, these procedures (tumour debulking and laminectomy for neural relief) significantly compromise spinal integrity, requiring subsequent spinal fixation to restore spinal stability. At present, the established gold standard for spinal stabilization is a long-segment fixation (*Lf*), involving the placement of pedicle screws two levels above and below the decompressed vertebra [4]. This surgery ensures greater mechanical support and stiffness but comes with notable drawbacks, including prolonged surgical time, extensive tissue incisions, and increased blood loss. Furthermore, with improved cancer treatments extending patients’ life expectancy, failure rates of these constructs have risen. Cai et al. [5], conducted a meta-analysis highlighting a hardware failure rate exceeding 10%, primarily due to pedicle screws mechanical complications, such as fatigue failure or dislodgement in the bone. Then, achieving less invasive and durable alternatives is becoming more and more a priority, supported by the improved minimally invasive surgical techniques [6–8]. The clinical paradigm of *Lf* is increasingly being questioned, with growing interest in short-segment fixations (*Sf*) involving only the levels adjacent to the decompression site [9–11]. However, *Sf* remains controversial among clinicians, as it is perceived to provide less stability and higher risks in cases of pedicle screw failure compared to *Lf*, where additional screws can better compensate for mechanical failures. Despite these concerns, Moussazadeh et al. [12] and Newman et al. [13] have reported that *Sf* has comparable revision rates to traditional *Lf* within the first few months post-surgery, demonstrating similar effectiveness in achieving primary stability. While longer-term data on *Sf* in oncologic surgeries after epidural cord compression are currently lacking, retrospective analyses in cases of thoracolumbar spine fractures suggest durable outcomes [14, 15].

From a biomechanical perspective, the practice of fixing multiple levels above and below the decompressed vertebra may excessively stiffen the spine-implant construct, leading to higher internal loads at the pedicle screw anchorages. Accordingly, Amankulor et al. [16] highlighted, in a retrospective study, that constructs spanning six or more levels are associated with a greater risk of implant failure. It becomes clear that better evidence on the loads to which pedicle screws are most frequently subjected over the course of postoperative life is needed.

As direct *in vivo* measurement of these loads is not feasible, numerical modelling provides a valuable alternative. Finite element (FE) multi-levels models have offered critical insight into deformation and stress distribution in spinal tissues and implant components across various decompression and stabilization strategies [17–19]. However, neither the kinetic nor the kinematic aspects of pathological models and fixation outcomes are typically validated. Additionally, due to computational costs, dynamic conditions and realistic screw geometries are rarely addressed. Single-level FE models are also used to examine factors such as pedicle screw geometry, insertion trajectory, as well as bone quality [20–22], but rely on simplified loading scenarios. Musculoskeletal multibody (MB) models offer a broader perspective by predicting postoperative spinal alignment, joint reactions, and muscle recruitment [23–25]. Despite this, such models often simplify instrumented spinal segments by rigidly constraining the involved intervertebral joints, making it impossible to accurately estimate internal spine-implant loads. Byrne et al. [26] stressed the importance of modelling intervertebral discs with six degrees of freedom and non-linear rotational stiffness to improve simulations reliability. Yet, many models do not address these aspects, neglecting facet joints and ligaments, and representing intervertebral joints as fixed centres of rotations with only non-linear rotational stiffness or as simple linear bushings [27].

This study proposes a combined non-linear rigid-flexible multibody approach to simulate spine-implant constructs, integrating the advantages of FE and MB dynamics. The aim is to investigate the internal reaction loads generated at the rod-pedicle screw anchorages in long-segment and short-segment fixations under fatigue-related displacement conditions. The underlying hypothesis is that long-segment constructs may increase mechanical risk factors for long-term failure of pedicle screws.

## Methods

### Creation of a Non-Linear Multibody Lumbar Spine Model

The MB model was developed by Adams software (v. 2017, MSC Software, Hexagon Corporate Services Ltd., UK) and represented the T12–S1 spinal segment. The vertebral geometry was derived from a Sawbones phantom (SKU3430). A previous study by Wang et al. [28] demonstrated the anatomical similarity of this synthetic spine model to human specimens, in terms of vertebral body height and pedicle height and width. Additionally, a volumetric correspondence within one standard deviation was confirmed when compared to human data [29]. The average density for cortical and cancellous bone was set to align statistically with that of an adult male (178 cm height, 75 kg weight). Further details are found in Table A.1 of the Supplementary Material.

**Table 1:** Input values for the considered implant rods.

Rod design variables	
Density $(\text{kg}/{\text{m}}^{3})$	1550
Diameter ($\text{mm})$	6
Young’s modulus $(\text{GPa})$	98
Curvature radius $(\text{mm})$	1000

**Fig. 1:**
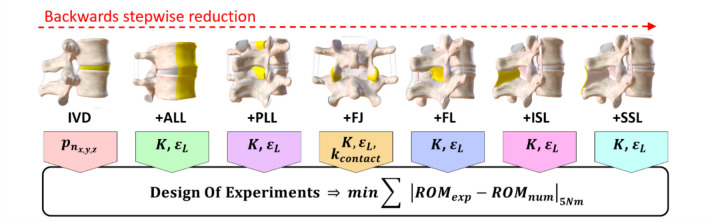
Flow of the backwards stepwise reduction. Starting from a configuration presenting only the intervertebral disc with its adjacent vertebrae, the entire FSU was built adding cumulatively all the passive elements. The figure shows all the studied configurations with the added element highlighted in yellow. For each step, the parameters in the coloured boxes were calibrated through the Design Of Experiments algorithm, minimizing the reported objective function.

The model was built based on the stepwise reduction of the L4–L5 Functional Spinal Unit (FSU) conducted by Heuer et al. [30], who characterized the passive FSU by progressively resecting all its passive elements. A backwards stepwise reduction was then implemented: starting from the final experimental configuration, which included only the two vertebrae and the interposed intervertebral disc (IVD), all intermediate configurations were recreated by progressively adding the spinal passive elements in reverse order of the experimental protocol (Fig. 1). Firstly, the behaviour of the IVD was modelled as follows (Eq. 1):

1 $$ \begin{aligned} \left[ {\vec{F}, \vec{M}} \right]^{T} & = \left[ {\begin{array}{*{20}c} {k_{x} \Delta s_{x} ,} & { k_{y} \Delta s_{y} ,} & {\begin{array}{*{20}c} {k_{z} \Delta s_{z} ,} & {\mathop \sum \limits_{n = 0}^{3} p_{n} {\Delta }\theta_{x}^{n} ,} & {\begin{array}{*{20}c} {\mathop \sum \limits_{n = 0}^{3} p_{n} {\Delta }\theta_{y}^{n} ,} & {\mathop \sum \limits_{n = 0}^{3} p_{n} {\Delta }\theta_{z}^{n} } \\ \end{array} } \\ \end{array} } \\ \end{array} } \right]^{T} \\ & \quad + \left[ {\begin{array}{*{20}c} {c\Delta \dot{s}_{x} ,} & { c\Delta \dot{s}_{y} ,} & {\begin{array}{*{20}c} {c\Delta \dot{s}_{z} ,} & {c_{t} \Delta \dot{\theta }_{x} ,} & {c_{t} \Delta \dot{\theta }_{y} \begin{array}{*{20}c} , & {c_{t} \Delta \dot{\theta }_{z} } \\ \end{array} } \\ \end{array} } \\ \end{array} } \right]^{T} \\ \end{aligned}, $$

where $\overrightarrow{F}$ and $\overrightarrow{M}$ represent the resulting force and moment vectors exerted by the IVD. A local reference system was defined, centred at the midpoint between the centroids of the upper vertebra’s inferior endplate and the lower vertebra’s superior endplate. The axes $x$, $y$, $z$ are aligned to the antero-posterior, medio-lateral, and superior–inferior directions. Translational forces are modelled as linear spring elements with stiffnesses coefficients ${k}_{x}$, ${k}_{y}$ , and ${k}_{z}$, with ${\Delta s}_{x}$, $\Delta {s}_{y}$, $\Delta {s}_{z}$ indicating the relative displacement of the vertebrae from their neutral stance along the IVD’s local axes. The rotational response is governed by a cubic polynomial, where ${\Delta \theta }_{x}$, $\Delta {\theta }_{y}$, $\Delta {\theta }_{z}$ correspond to relative rotations in lateral bending, flexion–extension, and axial rotation. A linear damping contribution is in all directions, with $c$ as the translational damping coefficient (5.8 N s/mm), and ${c}_{t}$ as the rotational damping coefficient (40.14 N mm s/°). Parameters used for IVD are detailed in Table A.2 of the Supplementary Material.

**Table 2:** Input values for the flexible connectors equivalent to the pedicle screws for each instrumented vertebra

Pedicle screw design inputs	
L1 level $(\alpha ,\varphi )$*	12°, 5°
L2 level $(\alpha ,\varphi )$	6°, 10°
L4 level $(\alpha ,\varphi )$	10°, 25°
L5 level $(\alpha ,\varphi )$*	12°, 25°
${k}_{x}$, ${k}_{y}$ $(\text{N}/\text{mm})$	5542
${k}_{z}$ $(\text{N}/\text{mm})$	Rigid
${k}_{Tx}$, ${k}_{Ty} (\text{Nm}/\text{rad})$	415.6
${k}_{Tz} (\text{Nm}/\text{rad})$	Rigid

Sagittal symmetry was considered for each instrumented vertebra. Stiffnesses directions are the following: $z$, parallel to the pedicle screw axes, $y$, perpendicular to the corresponding cross-section of the rod; $x$, mutually perpendicular to the previous two

*Only for *Lf*

Five ligaments, namely, the anterior longitudinal (ALL), posterior longitudinal (PLL), flava (FL), interspinal (ISL), and supraspinal (SSL) ligaments, were modelled as pre-tensioned, non-linear spring elements in parallel with a damper. For each ligament, the piecewise function in Eq. (2) was implemented [31, 32]. The strain $\varepsilon$ of each ligament is calculated as the ratio of the distance increment between its attachment points during dynamic simulations to its rest length. The rest length was determined by evaluating the ratio of the initial distance between the ligament’s attachment points in the model to the experimentally measured pre-strains found in literature [33, 34]. $K$ represents a stiffness per unit strain, which was specifically calibrated for each ligament. $c$ is the damping coefficient (2.3 N s/mm), and ${v}_{rel}$ is the relative velocity of the ligaments’ attachment points during dynamic simulations. The term ${2\varepsilon }_{L}$ defines the strain threshold at which the ligament transitions to linear behaviour. Detailed parameters for all ligaments are detailed in Table A.3 of the Supplementary Material.

2 $$ F = \left\{ \begin{gathered} \varepsilon \le 0\quad 0 \hfill \\ 0 < \varepsilon \le 2\varepsilon_{L} \quad - 0.25K\frac{{\varepsilon^{2} }}{{\varepsilon_{L} }} - cv_{{{\text{rel}}}} \hfill \\ \varepsilon > 2\varepsilon_{L} \quad - K\left( {\varepsilon - \varepsilon_{L} } \right) - cv_{{{\text{rel}}}} \hfill \\ \end{gathered} \right. $$

**Table 3:** Moment required to achieve the same prescribed range of motion (22° flexion, 8° extension, 12° right lateral bending and 5° counterclockwise axial rotation) and the resulting ROM distribution of *Lf* and *Sf* compared with the intact physiological model.

	Flexion, 22°			Extension, 8°			Lateral bending, 12°			Axial rotation, 5°		
	Intact	*Lf*	*Sf*	Intact	*Lf*	*Sf*	Intact	*Lf*	*Sf*	Intact	*Lf*	*Sf*
Moment	1.5 Nm	9.8 Nm	4.2 Nm	1.3 Nm	4.6 Nm	2.4 Nm	1.5 Nm	10. 2Nm	3. 4Nm	3.3 Nm	4.7 Nm	5.0 Nm
T12–L1	2.9°	+ 169.0%	+ 51.9%	1.6°	+ 135.1%	+ 44.6%	1.6°	+ 265.7%	63.7%	0.7°	+ 157.3%	+ 194.5%
L1–L2	2.1°	− 93.0 %	+ 40.1%	1.5°	− 95.5%	+ 59.2%	1.6°	− 92.9%	87.9%	0.6°	− 68.2%	+ 40.1%
L2-L3	3.8°	− 94.4 %	− 98.5%	1.3°	− 90.9%	− 97.1%	2.5°	− 89.0%	− 98.7%	0.7°	− 37.4%	− 68.2%
L3-L4	3.8°	− 98.9 %	− 99.9%	1.4°	− 99.9%	− 99.7%	2.5°	− 109.5%	− 100.6%	1.1°	− 66.3%	− 82.7%
L4-L5	4.8°	− 96.8 %	+ 48.2%	1.1°	− 93.2%	+ 25.7%	2.4°	− 94.9%	+ 59.2%	1.1°	− 72.0%	+ 38.3%
L5-S1	4.6°	+ 196.8%	+ 62.7%	1.2°	+ 231.4%	+ 57.3%	1.5°	+ 292.0%	+ 68.0%	0.8°	138.0%	+ 48.1%

The facet joint complex (FJ), comprising the articular facets and capsular ligaments, was modelled using a contact plane embedded with one orthogonal and two transversal spring-dampers. The orientations of the planes representing the articular facets were derived from the “card angles” proposed by Panjabi et al. [35]. Initially, the right and left facet angles were averaged for each vertebra (maintaining the superior and inferior facets distinguished). Subsequently, for each facet joint, the angles of the involved vertebrae were further averaged (i.e. the inferior facet angles of the superior vertebra with the superior facet angles of the inferior vertebra). Symmetry with respect to the sagittal plane was assumed. The forces exerted by the facet joints, as shown in Eq. (3), include two perpendicular components lying within the contact plane, which represent the contribution of the capsular ligaments (modelled in the same way as other ligaments). Additionally, the relative displacement of the centroids of the articular facets along the perpendicular direction of the contact plane (${\Delta s}_{z}$) permitted to consider the compression of the cartilage coating the facets, represented through a frictionless contact formulation with stiffness coefficient ${k}_{contact}$ (${\Delta s}_{z}<0$), and the resistance to vertebrae separation acted by the capsular ligaments (${\Delta s}_{z}>0$). The parameters for the facet joints are detailed in Table A.4 of the Supplementary Material.

3 $$ \left[ {\vec{F}} \right] = \left[ {\begin{array}{*{20}c} { K\frac{{\varepsilon^{2} }}{{2\varepsilon_{L} }} - cv_{{{\text{rel}}}} ,} & {K\frac{{\varepsilon^{2} }}{{2\varepsilon_{L} }} - cv_{{{\text{rel}}}} ,} & {\begin{array}{*{20}c} {\left( {k_{{{\text{contact}}}} \Delta s_{z} } \right)^{3} \Delta s_{z} < 0} \\ {K\frac{{\varepsilon^{2} }}{{2\varepsilon_{L} }} - cv_{{{\text{rel}}}} \Delta s_{z} > 0} \\ \end{array} } \\ \end{array} } \right]^{T} $$

**Table 4:** Reaction loads transmitted to pedicles screws generated at the rod-pedicle screw anchorages

	Flexion		Extension		Right lateral bending		Counterclockwise axial rotation	
	*Lf*	*Sf*	*Lf*	*Sf*	*Lf*	*Sf*	*Lf*	*Sf*
*Pullout forces (N)*								
L1 level	− 4.4, − 7.3	–	− 26.9, − 20.6	–	− 20.67,4.8	–	− 28.6, − 30.2	–
L2 level	3.7, 5.9	− 1.23, − 1.35	39.3, 32.2	3.0, 7.7	1.0, 15.6	− 5.3, 3.4	− 40.6, 104.5	− 42.6, 27.2
L4 level	3.7, 16.0	4.4, 9.2	− 26.1, − 24.9	− 1.3, − 0.6	25.1, − 25.5	14.5, − 14.3	42.6, − 56.0	46.0, − 46.5
L5 level	49.3, 55.2	–	− 12.0, − 16.2	–	128.9, − 126.4	–	27.3, − 16.8	–
*Shear forces (N)*								
L1 level	207.8, 215.2	–	142.7, 151.8	–	379.1, 396.9	–	143.6, 100.2	–
L2 level	78.4, 66.5	27.0, 61.1	48.4, 53.8	20.8, 46.2	48.9, 50.2	72.7, 70.7	82.6, 53.9	32.5, 30.8
L4 level	93.6, 47.9	26.7, 60.5	58.6, 44.2	20.6, 45.7	8.3, 10.7	71.4, 69.5	21.7, 27.9	27.5, 24.0
L5 level	216.5, 223.8	–	152.8, 158.6	–	397.2, 409.2	–	74.7, 69.9	–
*Bending moment (Nm)*								
L1 level	0.42, 0.38	–	0.05, 0.04	–	0.05, 0.22	–	1.09, 0.53	–
L2 level	0.12, 0.21	0.10, 0.06	0.41, 0.36	0.07, 0.06	0.12, 0.11	0.02, 0.03	2.27, 1.54	1.45, 1.43
L4 level	0.21, 0.39	0.15, 0.18	0.65, 0.67	0.13, 0.12	0.67, 0.68	0.07, 0.07	2.67, 2.48	1.65, 1.65
L5 level	0.83, 0.80	–	1.10, 1.09	–	0.44, 0.46	–	1.59, 1.41	–

Data are reported for all the spinal motions both for the long-segment and short-segment fixations. For each level, the values are reported as “left anchorage, right anchorage”. Negative pullout forces correspond to compressive force on the screws. The bending moment is reported along the direction of the main motion of the implant-spine construct (medio-lateral axis for flexion and extension, antero-posterior for lateral bending and craniocaudal axis for axial rotation)

Parameters ${p}_{n}$(Eq. 1), $K$, ${\varepsilon }_{L}$ (Eq. 2), $K$, ${k}_{\text{contact}}$, and ${\varepsilon }_{L}$ (Eq. 3) were calibrated using a Design of Experiment approach which minimised the objective function defined as the sum of the deviation between the numerical and experimental ROM under flexion, extension, lateral bending, and axial rotation at 5 Nm (Fig. 1). Once the single L4–L5 FSU model was finalised, the facet joints and ligaments characteristics were extended to all the vertebral levels of the T12–S1 model. Concerning the IVDs, the ROM-Moment responses (Eq. 1) of the rest of the vertebral levels were scaled from the L4–L5 polynomial coefficients based on the meta-analysis by Zhang et al. [36].

The validation of the final T12–S1 model was performed exclusively against experimental studies that tested multi-level specimens under pure moments [37, 38], as this load condition allows for avoiding the influence of possible artefacts in lumbar specimen responses caused by different approaches in the boundary load application [39].

### Modelling of the Surgical Fixations

**Fig. 2:**
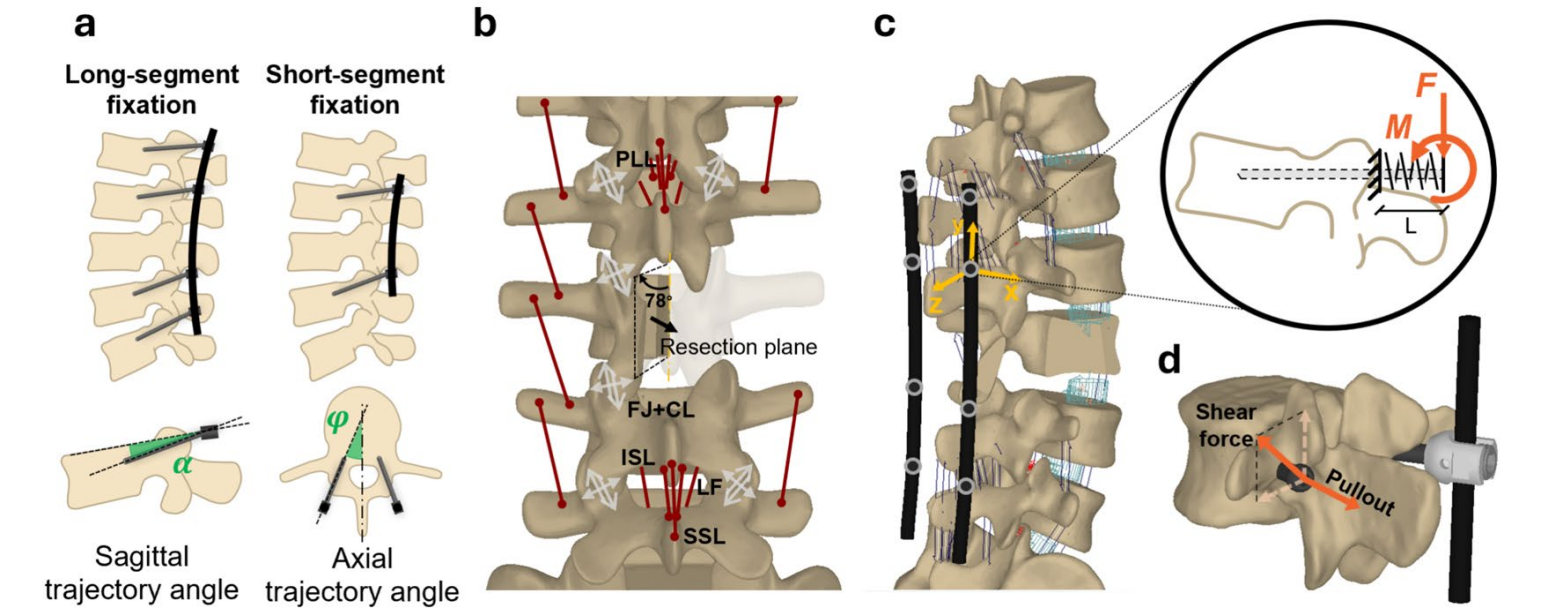
**a** Representation of the long-segment fixation and short-segment fixation and illustration of the pedicle screws insertion trajectory angles; **b** intermediate model reproducing the decompressed model; **c** Model reproducing the long-segment fixation; circle with grey outline along the rod corresponds to the rod-pedicle screw anchorages. An example of the local reference system describing the orientation of the pedicle screw is reported in yellow. The zoom highlights pedicle screw modelling represented as a bushing through cantilever beam theory (L: 15 mm); **d** loads transmitted to the pedicle screw at its anchorage: pullout force is aligned with the longitudinal axis of pedicle screws; shear force is the resultant of the forces applied on the plane orthogonal to the screw longitudinal axis. For each instrumented vertebra, both right and left anchorages were considered for the estimation of pedicle screw loads.

This study simulates a clinical scenario of a single-level L3 osteolytic metastasis and spinal cord compression, treated through posterior surgical decompression and resection of the right hemisome of the L3 vertebral body. Two fixation strategies were then modelled (Fig. 2a): a long-segment fixation (*Lf*), involving two spinal levels above and below the lesioned vertebra (L1, L2, L4 and L5), and a short-segment fixation (*Sf*) involving only the adjacent vertebrae (L2 and L4). The posterior decompression procedure also included facetectomy and the removal of LF, ISL, ISL, SSL at the L2–L3 and L3–L4 levels (Fig. 2b).

Both fixations were modelled in CFR-PEEK, given its growing interest in oncological spinal surgery due to advantages in post-treatment imaging and radiotherapy [40]. The rods were represented as flexible finite element bodies using the adds-on *Adams Flex* of Adams (MSC Nastran routine). Each rod was meshed with tetrahedral linear elements of 0.5 mm size, with straight edge shape. Their deformability was characterised using the modal analysis method, with the first 26 modes sufficient for capturing the system's dynamic behaviour. Flexible rods allowed for the simulation of the compliances of the spine-implant construct, enabling an accurate evaluation of load distribution [41]. Table 1 summarises the rods’ characteristics; the Young’s modulus was analytically derived from a four-point bending test on a commercial CFR-PEEK rod (Carbofix Orthopaedics Ltd, Herzliya, Israel) conducted with a universal testing machine Instron E3000 (Instron, Norwood, MA, USA) and subsequently verified by replicating the test also *in silico* (details are provided in the Supplementary Material, Sect. B. *Characterization of the CFR-PEEK rods*). The rods were symmetrically positioned at an average distance of 15 mm from the vertebral posterior pedicles, with their medio-lateral position aligned to mimic the traditional insertion trajectory of pedicle screws.

Pedicle screws were modelled as flexible connectors between the rods and the spine recurring to cantilever beam theory (Fig. 2c). Assuming complete osseointegration of the cortical bone with the screw threads, each pedicle screw was assumed as a short beam fixed at the insertion point in the vertebral peduncle and subjected to the loads transmitted by the rod at the other extremity. At each level, screws were oriented to mimic a traditional insertion trajectory (i.e. following the vertebral peduncle) defined by the sagittal trajectory angle $\alpha$ and the axial trajectory angle $\varphi$ [42] (Fig. 2a). The translational and rotational stiffnesses were calculated based on the properties of CFR-PEEK, with a polar moment of inertia derived from a cylinder of 15 mm length and 6.5 mm diameter. Table 2 resumes the design inputs for the pedicle screws.

The kinetic behaviours of the decompressed, *Lf* and *Sf* configurations were experimentally characterised in a prior study by the authors [43] using a T12–S1 biomimetic Sawbones. The changed L3 moment of inertia and the positions L2L3 and L3L4 centres of rotation were adjusted in accordance with the previous experimental data. Moreover, the experimental responses under flexion/extension, lateral bending, and axial rotation were used to validate the numerical models, by applying the same experimental boundary conditions. Details of the validation process are provided in the Supplementary Material (Sect. C *Validation of the surgical fixation models*). The internal reactions at each pedicle screw anchorages for both *Lf* and *Sf* were compared at parity of T12–S1 Range of Motion (ROM), set to one third of the average maximum healthy human T12–S1 ROM [44]: 22° flexion, 8° extension, 12° right lateral bending and 5° counterclockwise axial rotation (observing the segment from a top view) . These motions fall within the “Cone of Economy” [45], a volume in which the human body maintains postural balance with minimal muscular energy expenditure, relying primarily on the capacity of spinal passive elements. The loads experienced by the pedicle screws were described in terms of (1) pullout forces aligned with the screw’s longitudinal axis, (2) resultant shear forces acting on the plane orthogonal to the screw’s longitudinal axis, and (3) bending moments applied in the primary motion direction of spine-implant constructs.

## Results

### Validation of the Physiological MB Model

**Fig. 3:**
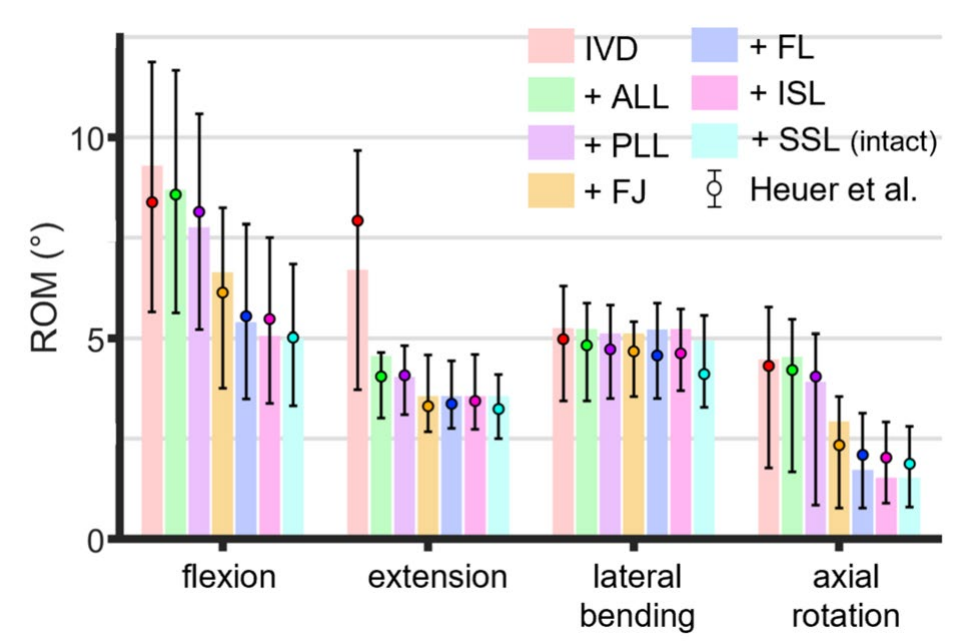
Comparison of the backwards stepwise reduction performed *in silico* with experimental data. Each label indicates the added element at each step. Experimental values are presented as the median ROM with minimum–maximum error bars.

Figure 3 presents the results of the calibration of the passive elements at each step of the backwards stepwise reductions. A strong agreement is observed between the *in silico* results and experimental data.

**Fig. 4:**
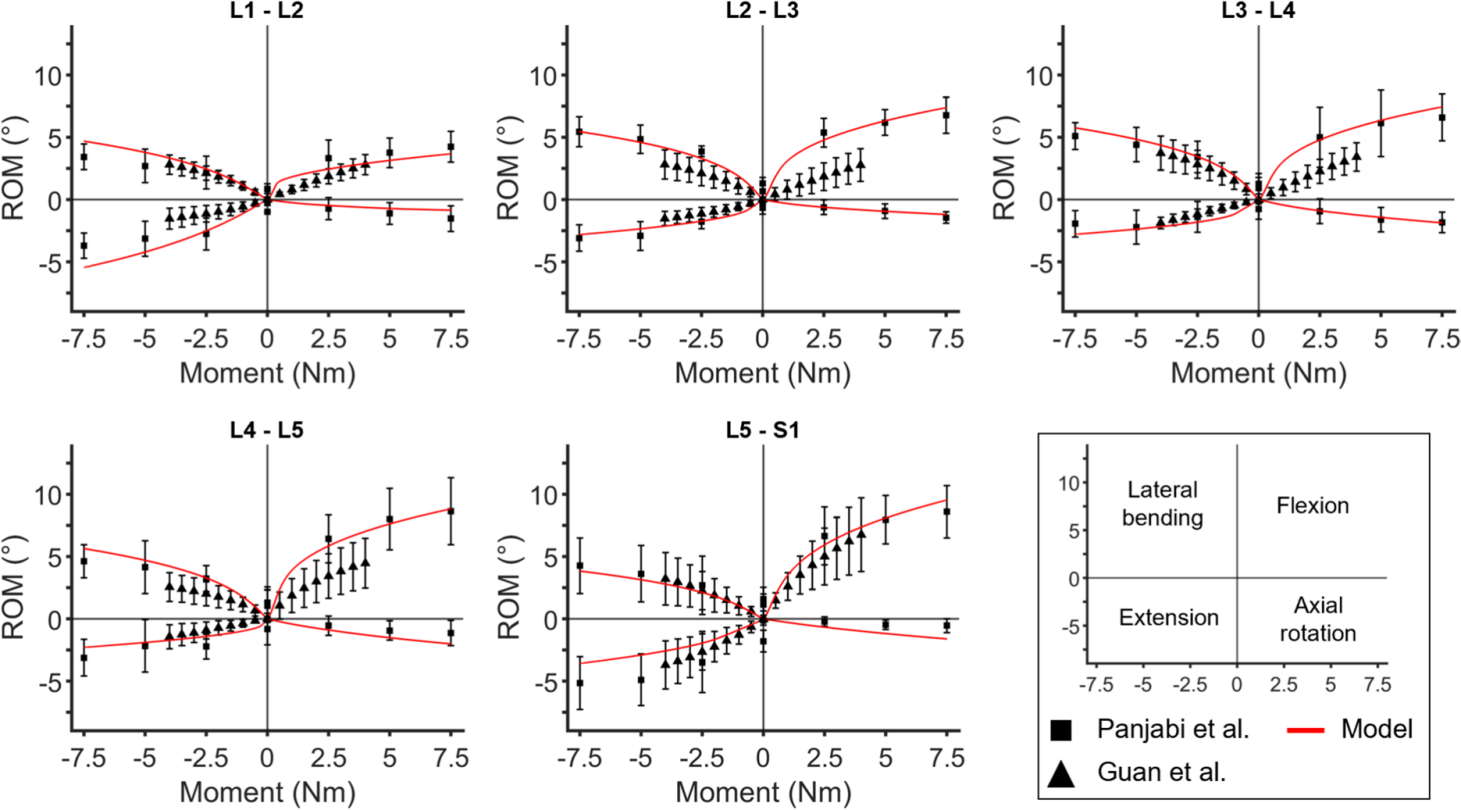
Moment-rotation behaviour of each intervertebral level in flexion (1st quadrant), extension (3rd quadrant), lateral bending (2nd quadrant), and axial rotation (4th quadrant) under ± 7.5Nm.

The T12–S1 multibody model also demonstrated good agreement with experimental data from the literature, despite the high variability typically observed in such experimental results. To ensure the most reliable validation, the model was compared against moment-rotation curves for each segmental level in flexion–extension, lateral bending, and axial rotation. Figure 4 highlights that the model not only replicates the overall non-linear behaviour of the spine across all directions but also accurately captures the responses at each intervertebral level (minimum coefficient of determination, ${R}^{2}$: 0.78 for L2–L3 in flexion–extension, 0.88 for L5–S1 in lateral bending, and 0.86 for L5–S1 in axial rotation).

### Reactions at the Rod-Pedicle Screw Anchorages

Details on the validation of the decompressed model, and the long-segment and short-segment fixations are provided in the Supplementary Material, Sect. C. *Validation of the surgical fixation models*. In all the spinal stances, *Lf* requires the application of greater loads to move within the Cone of Economy. Table 3 reports the spinal segment stiffening effect of the T12–S1 segment caused by the two fixation methods compared to the intact segment (i.e. a greater moment is needed to achieve the same prescribed range of motion), and the distribution of ROM across the vertebral levels.

**Fig. 5:**
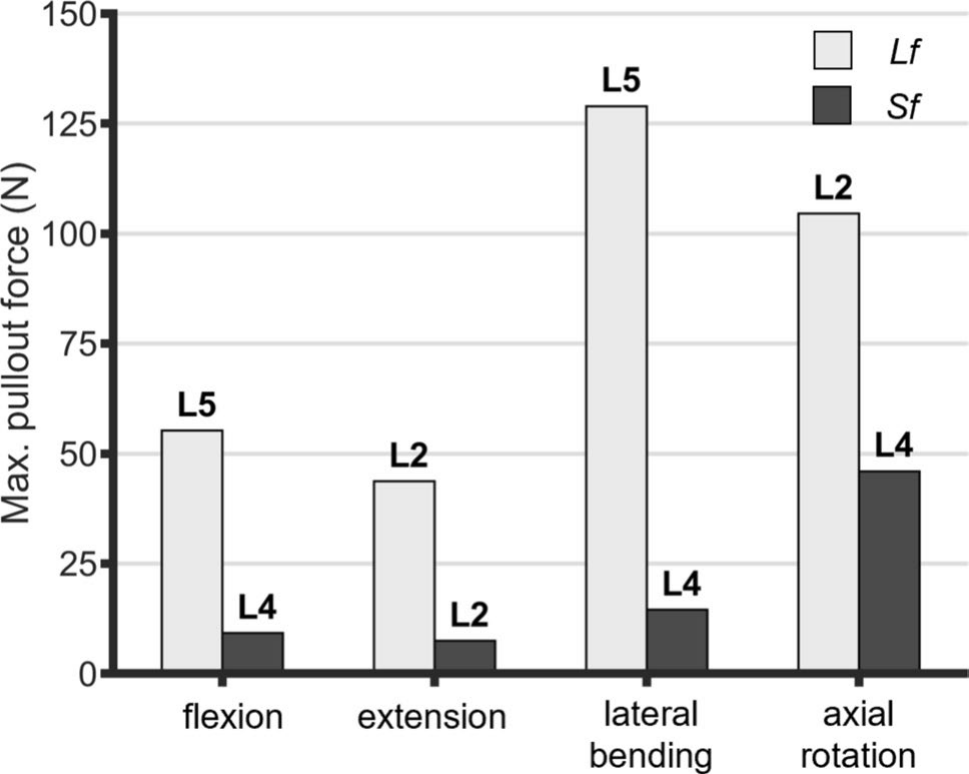
Maximum pullout force generated at the rod-pedicle screw anchorages during flexion, extension, lateral bending, and axial rotation. The labels indicate the vertebral level whose pedicle screws are majorly loaded.

Table 4 summarises the reaction loads transmitted to pedicle screw anchorages at the end of all motions. Figure 5 shows the maximum pullout forces and the level at which it is generated. The maximum pullout forces always remain within 150 N. *Sf* reduces the maximum pullout forces during all the motions: − 83.3 % in flexion, − 82.8 % in extension, − 88.7 % in lateral bending, − 56 % in axial rotation. During sagittal bending, intra-level screws experience similar loads (max. deviation intra-level ~ 10 N). Specifically, during flexion, pullout forces are generated at the L2, L4, L5 levels, while opposing compressive forces occur at the L1 level. In flexion, the implant resists to the extension of the lordotic curvature, which would otherwise result in a caudal divergence between the rod and the spinal segment. In the case of *Sf*, only the screws at L4 are slightly subjected to pullout forces which are comparable in magnitude to those observed at the least loaded level (L2) in *Lf* ( 9.2 *vs* 5.9 N, Table 4). In extension, only screws at L2 are subjected to pullout forces, with all the level subjected to higher compressive forces. During lateral bending and axial rotation, the forces acting on the right and left pedicle screws at each level are directed in opposing directions. In lateral bending, for both *Lf* and *Sf*, pullout forces are generated at the screw anchorages positioned on the rod on the bending side, above the decompression, and on the rod on the counter-bending side, below the decompression. Although the distribution pattern is similar, the maximum pullout in *Lf* reaches 128.9 N, whereas in *Sf*, the forces are nearly negligible (14.5 N, − 89%). Similar patterns are observed during counterclockwise rotation, where pullout forces are generated above the lesion on the rod that follows the rotation (right side for the counterclockwise rotation), and below the lesion on the rod pushed by the vertebral motion.

**Fig. 6:**
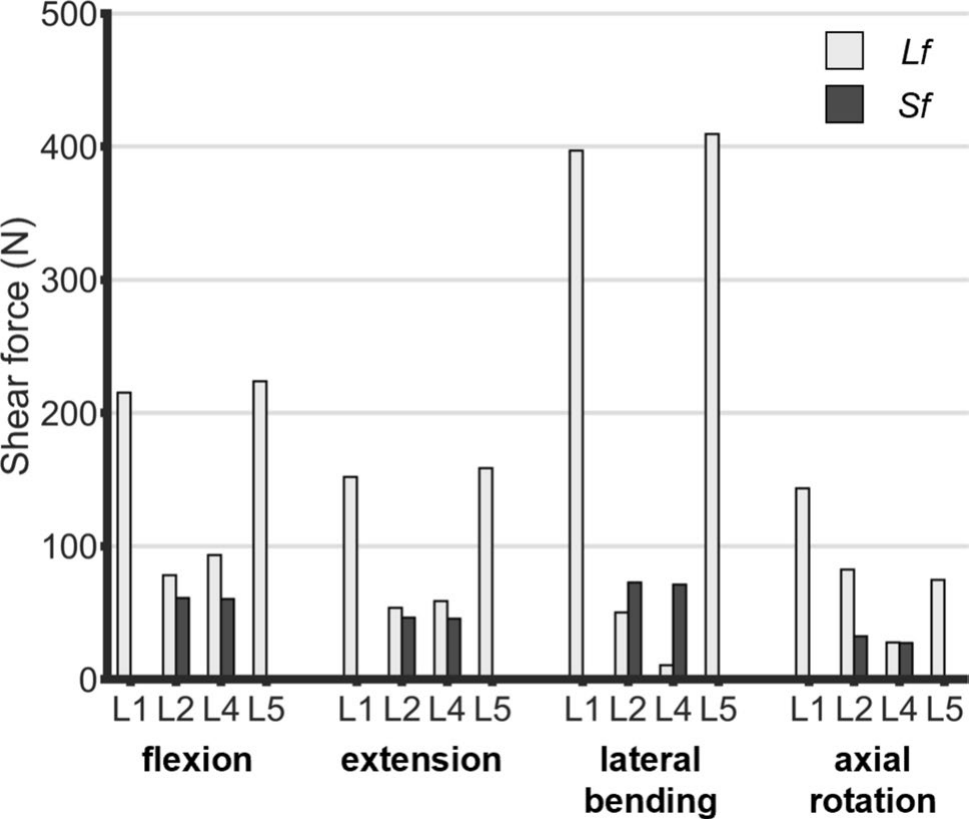
Shear forces applied at the rod-pedicle screw anchorages to the pedicle screws during flexion, extension, lateral bending, and axial rotation. The maximum force between the left and right anchorage is reported for each level.

Figure 6 shows the shear forces transmitted at the rod-pedicle screw anchorages. These loads are notably greater than pullout forces. During flexion and extension, *Lf* exhibits peaks at both extremity anchorages, reaching more than 200 N in flexion (over 6 times the pullout forces) and 150 N in extension (over four times the pullout forces). In contrast, *Sf* reveals a uniform load distribution, reduced by more than 70% in both flexion and extension compared to *Lf*. Lateral bending emerges as the most critical motion, inducing the highest shear forces. Long rods cause a heterogeneous distribution of the shear forces along the pedicle screws at different levels: central screws are largely shielded experiencing negligible forces (less than 50 N) at the expense of distal screws which are overloaded with forces up to 400N. Conversely, *Sf* achieves more balanced and contained shear forces of ~ 70N. In both cases, intra-level screws are subjected to shear forces with similar magnitudes (maximum difference always below 20 N). Finally, although axial rotation entails the lowest shear forces, it is the only motion which arises significant bending moments at the anchorages. In both *Lf* and *Sf*, all pedicle screws experience bending moments exceeding 1 Nm, with slightly greater variability in *Lf* (1.69 ± 0.73 Nm) compared to *Sf* (1.55 ± 0.12 Nm). The highest bending moments are observed at the L4 level (the inferior adjacent level of the lesion) with values of 2.6 Nm in *Lf* and 1.6 Nm in *Sf*.

## Discussion

### The Multibody Modelling Approach

This study evaluated how internal reactions developed at the rod-pedicle screw anchorages change between a long-segment and short-segment fixation for the stabilization of lumbar spine after a decompression surgical treatment.

To achieve this, a non-linear multibody model was previously validated using experimental data from physiological *ex vivo* lumbar segments. The designed multibody rigid model successfully caught spinal non-linearity. Only a few studies express the non-linear rotational behaviour of IVDs. Abouhossein at al. [46] used a series of B-splines; Rupp et al. [47] and Malakoutian et al. [48] integrated non-linear behaviour in a multibody model only in sagittal bending, limiting the applicability of their model. Wang et al. [49] presented a non-linear FSU stiffness, through an integrative formula obtained from single FSU experimental tests. This study proposes cubic polynomial stiffnesses for flexion, extension, lateral bending, and axial rotation, offering an effective and balanced strategy between implementation complexity, modelling efficacy, and computing costs. Furthermore, this study also specifically addressed further kinetic validation of both the spinal condition before the insertion of the implant, and the spinal fixations, which is an often-overlooked step in the numerical studies investigating spinal postoperative outcomes [50]. Integrating deformability in rigid multibody modelling allowed for the inclusion of rod flexibility, enabling the simulation of postoperative outcomes within a multibody environment, where fixations are typically modelled by rigidly constraining all involved vertebrae. The combined rigid-flexible approach also facilitated the estimation of anchorage loads under dynamic conditions with significantly reduced computational times (order of minutes), in strong contrast to the demands of FE models. However, it is important to keep in mind that this approach does not replace FE models, instead it lays the groundwork for future parametric studies and the enrichment of unidirectional workflows where reaction loads estimated via multibody analysis are transferred as boundary loads to FE model. This would allow for more realistic load simulations with greater precision also in the load transfer locations.

### The Choice of the Small-Displacement Domain

Several factors justify the decision to conduct the analysis within a small-displacement domain, ensuring that the T12–S1 ROM remains within the “Cone of Economy” volume.

Firstly, the defined ranges of motion were consistent with postoperative lumbar mobility, which is heavily restricted by the fixations. The moments required to achieve the investigated motions (Table 3) resulted realistic, not exceeding 10 Nm, a value considered as the reliable limit of the moment that musculature can exert on the lumbar segment. Secondly, remaining in a small-displacement domain, close to neutral posture, permitted to understand the loads transmitted to the screws, where repetitive loading and unloading cycles take on the characteristics of fatigue loading. These loads cannot be entirely avoided except in cases of prolonged bed rest. Indeed, the primary cause of pedicle screw loosening has been attributed to continuous cyclic loads persisting over time, rather than isolated, sporadic, high loads, which, conversely, pose a greater risk of implant breakage— an event reported to be much rarer.

Although the evaluation of the loads transmitted to pedicle screws can be extended to any surgical application involving posterior spinal fixation (i.e. thoracolumbar fractures), this study is particularly relevant to oncologic surgery. Indeed, advances in cancer treatments have significantly increased patient’s life expectancy, and ensuring greater implant durability becomes particularly vital as the frailty of oncological patients makes revision surgery both unsustainable and highly hazardous.

### Long vs. Short-Segment Fixation

Despite the small ROM investigated, the loads on pedicle screws varied significantly between long-segment and short-segment fixations. Estimating these loads revealed an effective way for understanding the impact of fixation length on the spine-implant construct, providing insights into how these loads are distributed in dynamic conditions, and identifying the most critical screw anchorages. This analysis also contributes to explaining the increasing number of failures observed in clinical practice in recent years.

Regarding pullout forces, while *Lf* entails higher forces, the estimated magnitudes always remain within normal operating conditions avoiding hazardous loads. Wu et al. [51] experimentally measured pullout strengths of three commercial pedicle screws with three insertion strategies during cyclic loading, reporting all values exceeding 1000N. The predicted pullout forces in this study were consistently below this threshold, with a minimum safety coefficient of 8.6 for *Lf*.

Conversely, shear forces emerged as the most critical loads, showing the greatest differences between the two fixation types. In detail, *Lf* resulted in heterogeneous load distribution, with screws adjacent to the lesion being extremely shielded, at the expense of distal screws which were subjected to high forces comparable to experimental fatigue strength reported in literature: Viezens at al. [52] reported critical forces of 300 and 454 N for standard screws and screws with larger diameters causing loosening. Weiser et al. [53] compared fatigue strength of standard and cortical threaded screws applying shear forces at the head of pedicle screws, finding values ranging from 250 to 330 N for bone mineral density > 120 mg/cm^3^.

The similarity between our findings and experimental data highlights that increasing the number of fixed levels triggers hazardous shear reactions at the pedicle screw anchorages, potentially leading to fatigue failures. These findings evidence that *Lf* ultimately increases the risk of biomechanical failure of the implanted pedicle screws in the long term.

At present, pullout tests remain the most common method for experimentally assessing the mechanical resistance of pedicle screws [51], despite shear forces and bending moments being the most hazardous loads for a screw. However, a growing number of experimental studies are integrating multicomponent loading scenarios, as these are considered more appropriate for simulating *in vivo* screw loosening [54, 55]. Our findings provide further evidence of the importance of introducing more complex loading scenarios to better simulate the forces experienced by screw anchorages. This is particularly relevant as screw heads are subjected to significant shear forces and bending moments, even within a small ROM. Finally, retrospective clinical studies [56, 57] have observed correlation between pedicle screw loosening and bone microfractures caused by excessive loadings or failed osteointegration due to insufficient load transfer to the bone tissue, both conditions emerged also in the *Lf* model simulations.

Some limitations of this study need to be addressed. A simplified clinical case was modelled, assuming a hemisome lesion involving only the L3 vertebra and subsequent surgical decompression, which resected half of the L3 vertebral body. However, vertebral metastases can present as more extensive lesions spreading along the spinal segment. This modelling choice was made to align with available experimental data, enabling direct validation of postoperative outcomes. Additionally, no modifications were applied to the vertebral body to represent the pathological model, despite cancer cells alter bone structure. Significant efforts have been made to understand and experimentally characterize metastatic vertebral bodies in terms of bone mechanical characteristics and microstructure [58, 59]. However, transitioning these insights to *in silico* environments remains a challenge, with only a few FE studies proposing specific constitutive laws for metastatic lesions in the vertebrae or modelling variations in bone mineral density [60, 61]. Finally, Bokov et al. [57] identified risk factors for pedicle screw failure, including morphological characteristics such as bone density (e.g. osteoporosis, dimensions of metastatic lesions) and patient-specific spinal curvature (e.g. variations in lumbar lordosis or thoracic kyphosis), as well as surgical factors, like the choice of screws, their insertion accuracy, the curvature of the rods, and their relative position and orientation with respect to the spinal curvature. While this study did not specifically investigate these factors, the combined rigid-flexible multibody modelling approach employed here is well suited for such parametric analyses, offering a robust framework to examine the complex force interactions between the spinal column and fixation devices under dynamic loading conditions.

## Conclusions

A T12–S1 MB model was developed able to capture the non-linear behaviour of intervertebral discs, ligaments, and facet joints. The proposed constitutive laws are readily applicable to musculoskeletal models. Long-segment and short-segment fixations following L3 decompression were simulated to investigate how the number of vertebral levels involved in fixation influences the loads on pedicle screw anchorages. The implant-spine constructs were analysed within a small-displacement domain to estimate the more frequent load exchanges generated by their dynamics, which could potentially lead to long-term fatigue failure risks. Our findings showed that while long-segment fixations can rely on a greater number of pedicle screws to maintain spinal stability in the event of screw failure, they may increase the risk of such failure due to shear forces comparable to experimental fatigue at the screw anchorages or severe load shielding. These results align strongly with recent clinical studies and stress the importance of re-evaluating current clinical paradigm—not only with respect to primary stability but also considering long-term effects in light of the increasing life expectancies of cancer patients. Short-segment fixations could be a viable alternative when long-segment fixation is not strictly required, particularly in the absence of severe anterior instability, reducing the biomechanical risks associated with fatigue failure. However, further patient-specific numerical investigations and clinical studies with both short- and long-term follow-up are essential to reinforce these findings and support the surgical stabilization after epidural cord decompression.

## Summary

The increasing cancer burden and the improved oncologic care have resulted in a growing incidence of vertebral metastasis and its related complications such as spinal cord compression which often requires surgical intervention and the stabilization with posterior pedicle screw fixation.

The current gold standard stabilization consists in fixing multiple levels above and below the lesion to provide supportive rigidity. This approach is considered more conservative, as the presence of a greater number of screws can compensate for the loosening of one. However, this study assumes that increasing the number of fixed levels could, in turn, trigger mechanical risk factors for pedicle screws failure.

Before investigating postoperative outcomes, a new T12–S1 multibody spinal model was designed to be valid in all the anatomical planes and be able to return the non-linearities of all spinal passive elements. Particularly, the rotational response of the intervertebral disc was reproduced through a cubic polynomial expression. Ligaments were expressed as a pre-tensioned non-linear spring in parallel to a damper, while facet as a contact function integrated with spring-damper. The model was built through a backwards stepwise reduction strategy integrated with an optimization routine.

Starting from this model, a L3 decompressed condition was realized. Both short-segment and long-segment fixations were then modelled. The rods were represented as flexible finite element-based bodies whose deformability was described based on the modal analysis method; a four-point bending test on commercial CFR-PEEK rods was used to characterize them. Pedicle screws were modelled recurring to cantilever beam theory as flexible connectors between the rods and the vertebrae and oriented to mimic a traditional insertion trajectory. The kinetic behaviours of the decompressed spine and both fixations were also validated. Finally, the reactions at the rod-pedicle screw anchorages were estimated in terms of pullout, shear forces, and bending moment. The models were tested under 22° flexion, 8° extension, 12° lateral bending, and 5° axial rotation, keeping the range of motion within the “Cone of Economy”: indeed, this domain is characterized by a relevant contribution of passive elements and lowest muscular activity, and, at the same time, it is associated to postural balance, permitting to explore loads which cannot be avoided during postoperative life even maintaining reduced mobility.

The estimated pullout forces always remained confined within safe values (< 150N) in both fixations. Conversely, shear forces registered higher magnitudes, particularly in lateral and sagittal bending. Long-segment fixation showed a heterogeneous distribution of the shears along the implant, reporting shielded screws at the middle anchorages and overloaded ones at the extremities with forces greater than 300N, comparable with screws experimental fatigue strength. Axial rotation was the only motion with bending moment greater than 1Nm (maximum value of 2.6 Nm in long-segment fixation *vs* 1.6 Nm in the short one).

In conclusion, this study proves that an increase in the number of fixed levels leads to higher loads transmitted to pedicle screws, potentially accelerating adverse fatigue issues.

## Supplementary Information

Below is the link to the electronic supplementary material.

Supplementary file1 (PNG 848 KB)

Supplementary file2 (PNG 717 KB)

Supplementary file3 (PDF 1101 KB)

## Abbreviations

*Lf*: Long-segment fixation
*Sf*: Short-segment fixation
FSU: Functional spinal unit
ROM: Range of motion
CFR-PEEK: Carbon fibre-reinforced PEEK
IVD: Intervertebral disc
ALL: Anterior longitudinal ligament
PLL: Posterior longitudinal ligament
FL: Flava ligament
ISL: Interspinal ligament
SSL: Supraspinal ligament
FJ: Facet joint
