# Supplementary figures and images for: Combined Rigid-Flexible Multibody Analysis Reveals Reduced Pedicle Screw Loads in Short-Segment Fixation for Decompressed Lumbar Spine Stabilization
Source: Ann Biomed Eng. 2025 Mar 13;53(5):1257–69. doi: 10.1007/s10439-025-03706-1 (PMC12006242; doi:10.1007/s10439-025-03706-1)

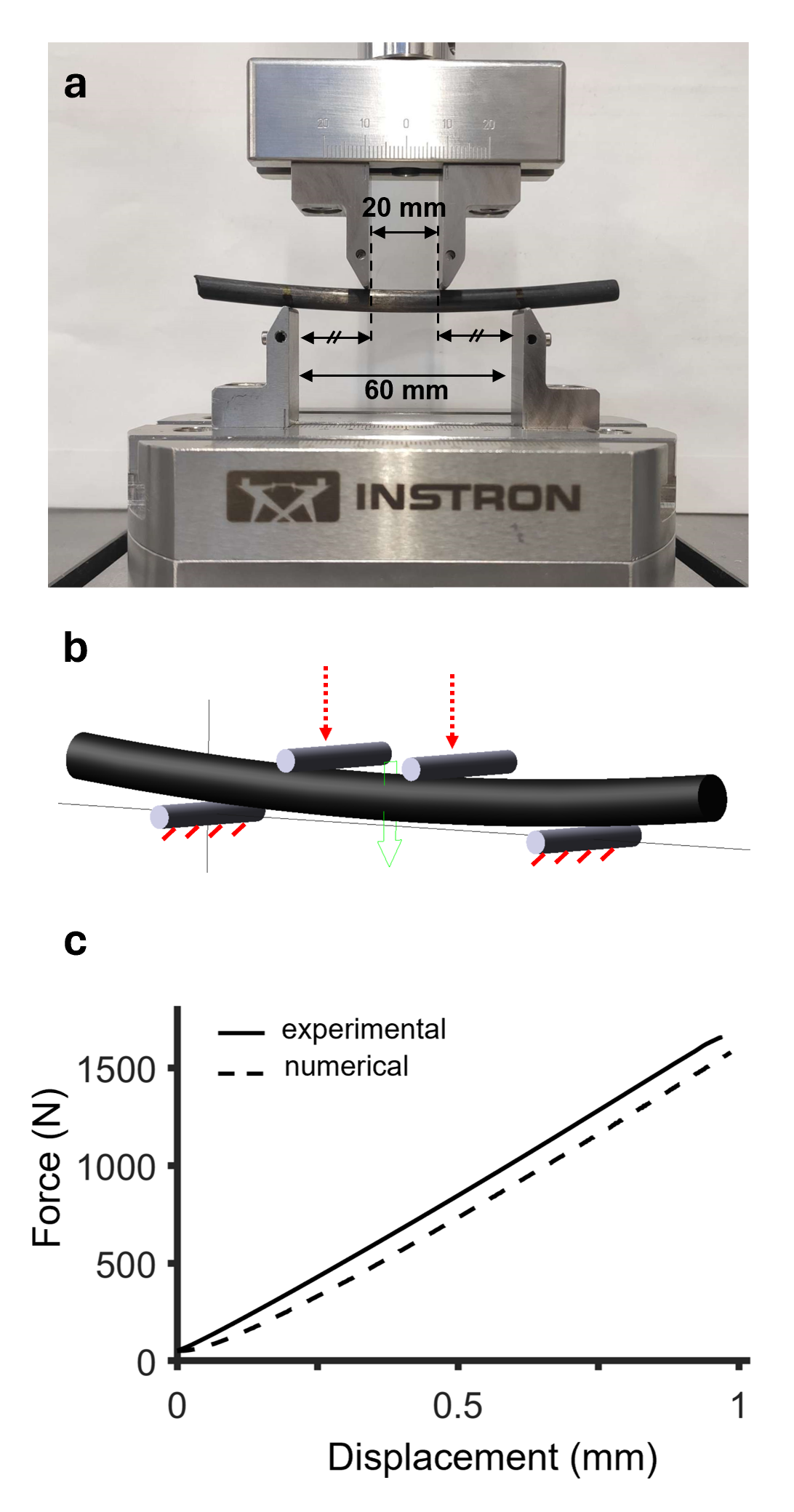

Supplement: Supplementary file 1 — Supplementary file1 (PNG 848 KB) [file 10439_2025_3706_MOESM1_ESM.png]

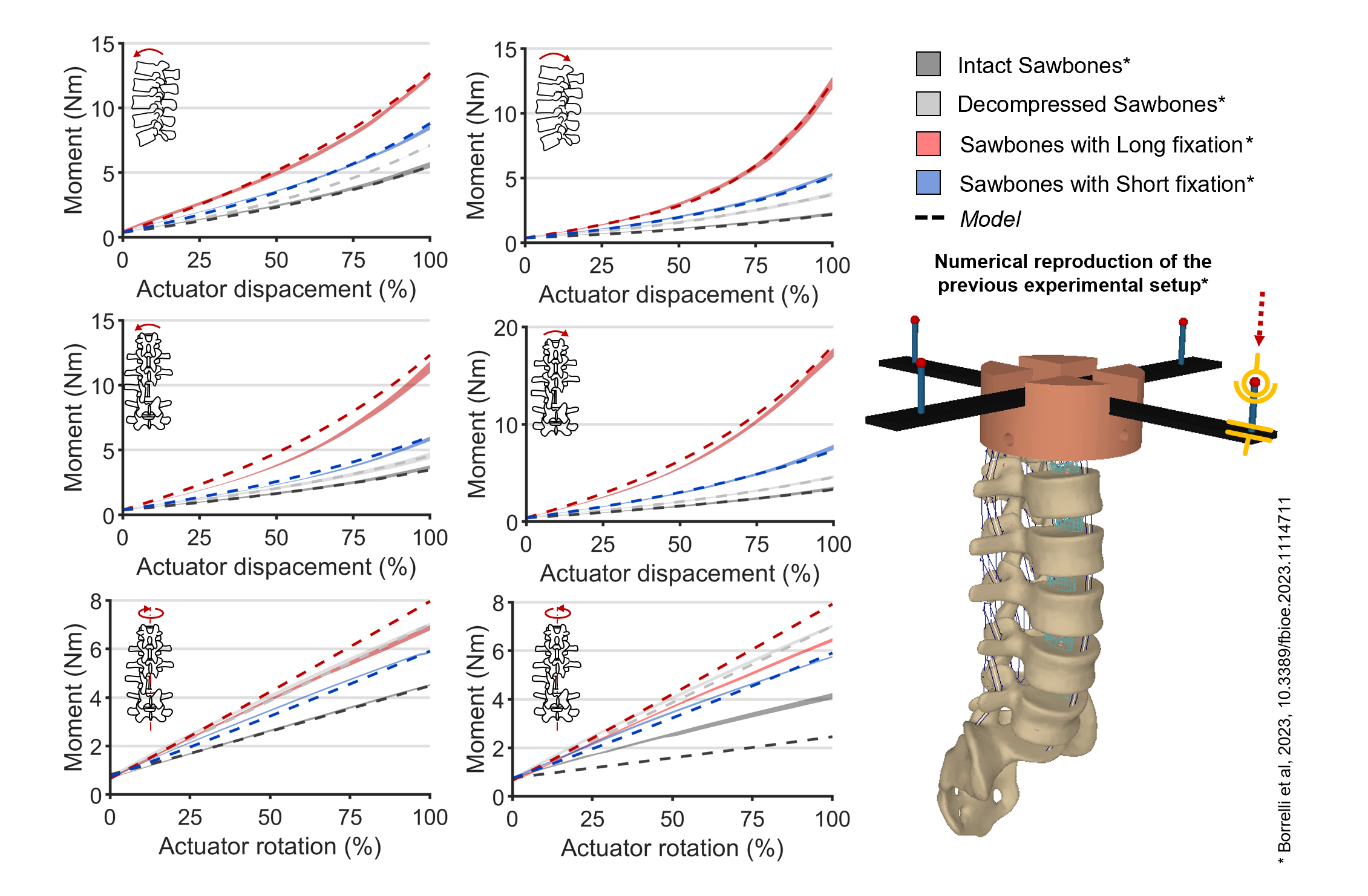

Supplement: Supplementary file 2 — Supplementary file2 (PNG 717 KB) [file 10439_2025_3706_MOESM2_ESM.png]
