# Supplementary material for: Combined Rigid-Flexible Multibody Analysis Reveals Reduced Pedicle Screw Loads in Short-Segment Fixation for Decompressed Lumbar Spine Stabilization
Source: Ann Biomed Eng. 2025 Mar 13;53(5):1257–69. doi: 10.1007/s10439-025-03706-1 (PMC12006242; doi:10.1007/s10439-025-03706-1)
Supplement: Supplementary file 3 — Supplementary file3 (PDF 1101 KB) [file 10439_2025_3706_MOESM3_ESM.pdf]

Supplementary materials

COMBINED RIGID-FLEXIBLE MULTIBODY ANALYSIS REVEALS REDUCED PEDICLE SCREW LOADS IN SHORT-SEGMENT FIXATION FOR DECOMPRESSED LUMBAR SPINE STABILIZATION

Simone Borrelli[1,2], Giovanni Putame[1,2], Stefano Marone[3], Andrea Ferro[3], Alberto L. Audenino[1,2], Mara Terzini[1,2].

1. Polito^{BIO}Med Lab, Politecnico di Torino, Turin, Italy.
2. Department of Mechanical and Aerospace Engineering, Politecnico di Torino, Corso Duca degli Abruzzi 24, 10129, Turin, Italy.
3. Oncologic Orthopaedic Surgery Division, CTO Hospital – Città della Salute e della Scienza di Torino, Turin, Italy.

A. Reproducibility of the physiologic MB model

Details on the vertebrae's centres of mass and inertia are already displayed and available [1]. The correspondence of the vertebrae between the model human data was assessed in terms of the vertebral body volume [2] and the vertebral mass was imposed regressing the equation proposed by Avtandilashvili and Tolmachev [3] (58 years-old, 75 kg weight, 178 cm height) and computing the percentage of the skeleton weight of each vertebra [4].

$$\boldsymbol{W_{skelet}} = \boldsymbol{-0.25 + 0.046 * Height\ (cm) + 0.036 * Weight\ (kg) - 0.012 * Age(y)} = 9.942\ kg$$

Table A.1 Correspondence of anatomical data with the geometrical input parameters of the used model.

	**Vertebral body volume (cm³)**		**% of body weight$|_{\mathrm{kg}}$, mass (g)**
	Human data	Model	Model
T12	27. 3 ± 6.7	22.5	0.42%, 41.8
L1	38.2 ± 9.3	35.4	0.50%, 49.7
L2	41.4 ± 7.9	36.7	0.59%, 58.7
L3	44.2 ± 10.1	38.6	0.66%, 65.6
L4	44.6 ± 9.9	40.0	0.67%, 66.6
L5	42.5 ± 10.1	41.8	0.68%, 67.6
S1	/	174.3	1.8%, 179.0

Table A.2: Input data for IVD modeling from T12-L1 to L5-S1 Functional Spinal Units (FSU) included.

**IVD**	T12-L1	L1-L2	L2-L3	L3-L4	L4-L5	L5-S1
k_x, antero-posterior stiffness (N/mm)	250.0	250.0	250.0	250.0	250.0	250.0
k_y, medio-lateral stiffness (N/mm)	250.0	250.0	250.0	250.0	250.0	250.0
k_z, cranio-caudal stiffness (N/mm)	1800.0	1800.0	1800.0	1800.0	1800.0	1800.0
Flexion-extension stiffness						
p_1, 1st order parameter $(Nmm/°)$	175.52	90.74	82.50	90.67	83.33	146.80
p_2, 2nd order parameter $(Nmm/(°)^2)$	-17.67	-16.23	-15.44	-20.54	-23.39	-9.68
p_3, 3rd order parameter $(Nmm/(°)^3)$	36.69	13.55	14.16	13.42	10.09	6.76
Lateral bending stiffness						
p_1, 1st order parameter $(Nmm/°)$	720.77	784.64	395.12	385.88	400.01	563.76
p_2, 2nd order parameter $(Nmm/(°)^2)$	0	0	0	0	0	0
p_3, 3rd order parameter $(Nmm/(°)^3)$	80.31	33.72	31.25	25.13	24.75	51.02
Axial rotation stiffness						
p_1, 1st order parameter $(Nmm/°)$	763.75	763.75	763.75	763.75	913.75	1363.75
p_2, 2nd order parameter $(Nmm/(°)^2)$	0	0	0	0	0	0
p_3, 3rd order parameter $(Nmm/(°)^3)$	20.24	20.24	20.24	20.24	20.24	20.24

Table A.3: Input data for ligaments modeling from T12-L1 to L5-S1 Functional Spinal Units (FSU) included.

**Ligament**	T12-L1	L1-L2	L2-L3	L3-L4	L4-L5	L5-S1
**Anterior longitudinal ligament (ALL)**						
$K\ (N)$	1396.1	1374.9	882.7	1676.2	1718.6	560.2
ε_0	0.136	0.136	0.136	0.136	0.136	0.136
ε_L	0.8	0.8	0.8	0.8	0.8	0.8
**Posterior longitudinal ligament (PLL)**						
$K\ (N)$	404	690.8	1478.6	428.2	1042.3	880.7
ε_0	0.088	0.088	0.088	0.088	0.088	0.088
ε_L	0.8	0.8	0.8	0.8	0.8	0.8
**Flava ligament (FL)**						
$K\ (N)$	89	84.6	92.3	126.9	100	74.9
ε_0	0.072	0.072	0.072	0.072	0.072	0.072
ε_L	0.7	0.7	0.7	0.7	0.7	0.7
**Interspinal ligament (ISL)**						
$K\ (N)$	69.5	57.5	55.2	104	50	93.7
ε_0	-0.058	-0.058	-0.058	-0.058	-0.058	-0.058
ε_L	0.8	0.8	0.8	0.8	0.8	0.8
**Supraspinal ligament (SSL)**						
$K\ (N)$	353	537.7	579.8	813.6	420.8	416.2
ε_0	-0.12	-0.12	-0.12	-0.12	-0.12	-0.12
ε_L	0.9	0.9	0.9	0.9	0.9	0.9

Table A.4: Input data for facet joints modeling from T12-L1 to L5-S1 Functional Spinal Units (FSU) included.

**Facet joints**	T12-L1	L1-L2	L2-L3	L3-L4	L4-L5	L5-S1
CA_x (°)	-79.5	-83.13	81.88	-78.98	-80.48	-76.3
CA_y (°)	±49.4	±56.3	±46.6	±35.4	±26.73	±37.86
ε_L	0.8	0.8	0.8	0.8	0.8	0.8
$k_{contact}$ (N/mm)	270.0	270.0	180.0	180.0	180.0	270.0
K (N)	4500	4500	4500	4500	4500	4500

B. Characterization of the CFR-PEEK rods

A CFR-PEEK was tested with a four-point bending test using the Instron E3000 linear-torsion universal testing machine (Instron Corporation, Norwood, MA, USA). Figure B.1a shows the setup and the obtained experimental curve maintaining only the loading phase. The test consisted of the application of a 1 mm vertical displacement by the two actuators 20 mm distant. The rod was placed on two supports that were 60 mm apart. The vertical displacement was applied at 5 mm/min rate to reduce any viscoelastic effects when an initial slight contact between the rod and the test machine was recorded by the load-cell (located in the upper part of the test machine). Starting from Newton's laws at the equilibrium, the Young's modulus has been determined as 98 GPa.

Furthermore, to verify the goodness of flexible modal approach to model the numerical rod, the experimental four-point bending test was also replicated in the numerical environment (Figure B.1b). The same boundary conditions were applied: two cylinders below the rod were fixed to the ground, while the testing machine was represented as two cylinders moving with the same rate as the experimental protocol. Contact was established between the various components. A satisfactory agreement emerged between the numerical curve and the experimental data with $R^2 = 0.91$ (Figure B.1c).

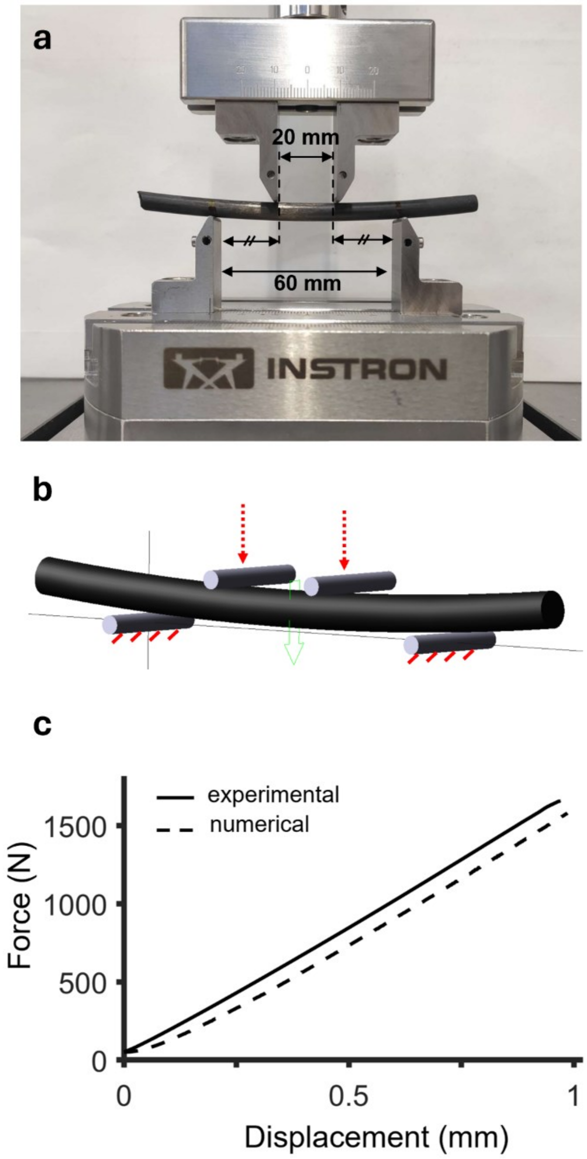

Figure B.1: a. The four-point bending test set up; b. The four-point bending test replicated *in silico*; c. Comparison between the experimental and numerical results.

C. Validation of the surgical fixation models

From an experimental perspective, the paucity of *in vitro* data describing the alteration of long-chain spinal stiffness and vertebral kinematics of pathological and instrumented spinal segments doesn't offer an exhaustive vision of altered biomechanical behaviour, leading in turn numerical models to be only validated based on physiologic responses.

To provide a data-driven validation of the models simulating the decompressed condition, and post-operative outcomes, this work relied on a previous experimental study conducted by the authors on a Sawbones phantom extending from T12 to the sacrum [6]. Briefly, the specimen was tested through a linear torsion testing machine (Instron E3000). Flexion/extension and lateral bending were applied through an eccentric load at T12 with the sacrum fully constrained, while axial rotation was applied by applying a torque to T12. Figure C.1 shows the numerical reproduction of the experimental setup. By applying the same

boundary conditions, the kinetic response (moment applied by the machine to achieve the imposed motion) recorded *in vitro* was compared with the results obtained *in silico* for the intact phantom (control), the decompressed condition and the long-segment and short-segment fixations.

Due to the stiffer and more linearized behaviour of the phantom, a specific calibration of the model parameters was necessary, achieving a control MB model representative of the Sawbones (C_{SAW}). To do that, linear rotational stiffness was added in all the intervertebral disc joints (2 Nm/° in sagittal and transversal axes and 1.500 Nm/° along the longitudinal axis). The decompression and the long-segment and short-segment fixations were then implemented starting from C_{SAW} and directly validated with the experimental data. The experimental set up was reproduced *in silico* with the same boundary conditions and load entities.

Figure C.1 reports the results of the validation, highlighting an extremely good estimation of the models, notwithstanding the small experimental deviations given by the high experimental reproducibility of the synthetic phantom. Furthermore, the asymmetrical behaviour of the decompressed segment recorded in lateral bending and axial rotation were faithfully returned by the numerical model. In axial rotation, the numerical model amplified the disparity between the two directions, with a greater flexibility, presumably due to the removal of L2-L3 and L3-L4 facet joints: if along the intact side, the numerical value is always within the experimental range, on the resected side, the rotational stiffness is majorly decreased (1.06 Nm/° *vs* 1.32 Nm/°).

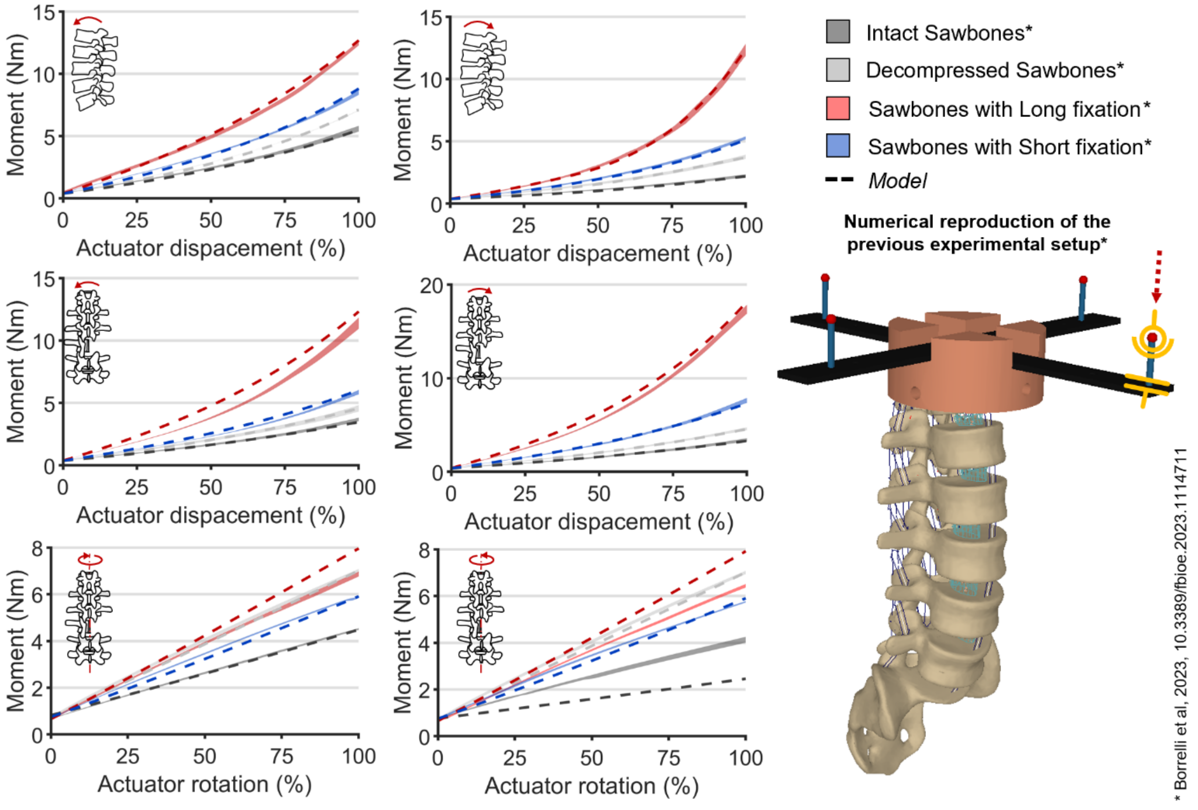

Figure C.1: Comparison of the intact (C_{SAW}), decompressed, *Lf* and *Sf* model with experimental data, according with the experimental tests previously attained [6]. On the right, the intact numerical model with the reproduction of the experimental setup. Flexion, extension, and lateral bending were performed through eccentric load.

References

[1] Borrelli, S., Putame, G., Terzini, M., Pascoletti, G. & Zanetti, E. Input data for the creation of a multibody lumbar model. *Mendeley Data* (2022).

[2] Limthongkul, W., Karaikovic, E. E., Savage, J. W. & Markovic, A. Volumetric analysis of thoracic and lumbar vertebral bodies. *Spine J.* **10**, 153–158 (2010). DOI: 10.1016/j.spinee.2009.11.018.

[3] Avtandilashvili, M. & Tolmachev, S. Y. Modeling the Skeleton Weight of an Adult Caucasian Man. *Health Phys.* **117**, 149–155 (2019). DOI: 10.1097/HP.0000000000000881.

[4] Lowrance, E. W. & Latimer, H. B. Weights and variability of components of the human vertebral column. *Anat. Rec.* **159**, 83–88 (1967). DOI: 10.1002/ar.1091590112.

[5] Panjabi, M. M. *et al.* Articular facets of the human spine:Quantitative three-dimensional anatomy. *Spine (Phila. Pa. 1976).* **18**, 1298–1310 (1993). DOI: 10.1097/00007632-199308000-00009.

[6] Borrelli, S. *et al.* Cross-link augmentation enhances CFR-PEEK short fixation in lumbar metastasis stabilization. *Front. Bioeng. Biotechnol.* **11**, 1–10 (2023). DOI: 10.3389/fbioe.2023.1114711.
